# Leveraging Symptom Search Data to Understand Disparities in US Mental Health Care: Demographic Analysis of Search Engine Trace Data

**DOI:** 10.2196/43253

**Published:** 2023-01-30

**Authors:** Ben Rochford, Sachin Pendse, Neha Kumar, Munmun De Choudhury

**Affiliations:** 1 School of Interactive Computing College of Computing Georgia Institute of Technology Atlanta, GA United States

**Keywords:** mental health, search engine algorithms, digital mental health, health equity

## Abstract

**Background:**

In the United States, 1 out of every 3 people lives in a mental health professional shortage area. Shortage areas tend to be rural, have higher levels of poverty, and have poor mental health outcomes. Previous work has demonstrated that these poor outcomes may arise from interactions between a lack of resources and lack of recognition of mental illness by medical professionals.

**Objective:**

We aimed to understand the differences in how people in shortage and nonshortage areas search for information about mental health on the web.

**Methods:**

We analyzed search engine log data related to health from 2017-2021 and examined the differences in mental health search behavior between shortage and nonshortage areas. We analyzed several axes of difference, including shortage versus nonshortage comparisons, urban versus rural comparisons, and temporal comparisons.

**Results:**

We found specific differences in search behavior between shortage and nonshortage areas. In shortage areas, broader and more general mental health symptom categories, namely *anxiety* (mean 2.03%, SD 0.44%), *depression* (mean 1.15%, SD 0.27%), *fatigue* (mean 1.21%, SD 0.28%), and *headache* (mean 1.03%, SD 0.23%), were searched significantly more often (*Q*<.0003). In contrast, specific symptom categories and mental health disorders such as *binge eating* (mean 0.02%, SD 0.02%), *psychosis* (mean 0.37%, SD 0.06%), and *attention-deficit/hyperactivity disorder* (mean 0.77%, SD 0.10%) were searched significantly more often (*Q*<.0009) in nonshortage areas. Although suicide rates are consistently known to be higher in shortage and rural areas, we see that the rates of suicide-related *searching* are lower in shortage areas (mean 0.05%, SD 0.04%) than in nonshortage areas (mean 0.10%, SD 0.03%; *Q*<.0003), more so when a shortage area is rural (mean 0.024%, SD 0.029%; *Q*<2 × 10^–12^).

**Conclusions:**

This study demonstrates differences in how people from geographically marginalized groups search on the web for mental health. One main implication of this work is the influence that search engine ranking algorithms and interface design might have on the kinds of resources that individuals use when in distress. Our results support the idea that search engine algorithm designers should be conscientious of the role that structural factors play in expressions of distress and they should attempt to design search engine algorithms and interfaces to close gaps in care.

## Introduction

### Background

Over the course of a given year, 1 in 5 US adults will experience mental illness [1]. This ratio is starker for young adults, with nearly 1 in 2 US adolescents experiencing mental illness within a given year [1]. Most people experiencing mental illness do not receive care, with only 46.2% of US adults experiencing mental illness receiving any form of care in 2020 [1]. Early signs suggest that the stress of coping with the COVID-19 pandemic [2] has caused an increase in the prevalence of serious mental distress [3] with longitudinal impacts that are not yet fully understood.

However, the sudden and widespread move toward remote work during the pandemic has also influenced the provision of care for mental illness—what Shore et al [4] call “the rapid virtualization of psychiatric care.” Given the requirements of social distancing and pandemic-incited isolation, an increasing number of individuals have turned to technology-mediated tools and resources to find help when in distress, including online support communities [5], helplines [6], resources recommended by search engines [7], teletherapy [8], and telepsychiatry [4], among other modalities. Recommendation algorithms that analyze individual language around mental health underlie how these tools suggest resources to people in need [9], including ways that may be opaque to those engaging with the technology- or algorithmically mediated support system [10].

As technology-mediated tools and resources expand access to care, structural and societal divides in American society are particularly important to study to ensure that inequities are not exacerbated by how algorithmically directed interventions are designed or deployed. Disparities in household income [11], care resources [12], race and ethnicity [13,14], sexual orientation [15,16], and gender identity [17] have all been demonstrated to have a substantial influence on how people experience mental illness and whether care is accessible. Much has also been written about the digital divide in the United States, with nearly 3 in 10 rural Americans without a broadband connection at home [18] and roughly 3 in 10 Black adults and 4 in 10 Hispanic adults without a broadband connection at home [19]. The sudden move to remote health care services during the COVID-19 pandemic made digital divides more sharply consequential [20], with some even arguing that they functioned as a social determinant of health [21,22] given that telehealth use was a primary means of care amid overburdened hospitals.

A similar divide exists in the availability of mental health care providers in the United States. In the United States, approximately 113 million people [23] live in mental health professional shortage areas (MHPSAs). MHPSAs are designated by the US Health Resources and Services Administration (HRSA) as facilities, population groups, and geographic areas in which there are “too few...mental health providers and services” to meet expected needs [24]. In this study, for brevity, we describe MHPSAs as “shortage areas” and areas that are not MHPSAs as “nonshortage areas.” Although it is well recognized that economic, social, and geographic disparities influence the accessibility and use of care in shortage areas, individual and community expressions of mental health in shortage areas are not as well understood or investigated. This lack of understanding is a significant gap in providing culturally competent care and can have a severe impact on the provision of care, particularly for those in geographically sparse or hard-to-reach areas [25,26].

One way in which people understand their experience of health and learn more about symptoms is via search engine queries and the subsequent resources recommended by the engines [27,28]. A Pew Research study conducted in 2013 [29] found that over 1 in 3 Americans search on the web for information about their health experiences, and nearly 8 in 10 of those who do search on the web for information and care begin their journey through a search engine. As traces of private engagements with a technology-mediated tool, search engine data can provide valuable insights into how individuals understand their own mental health and express it to others without the limitations of external social stigma [30].

### Objective

In this study, by leveraging deidentified and aggregated data from Google (Google LLC) searches regarding symptoms from 2017 to 2021 [31], we analyzed how differences in access to mental health resources relate with how individuals search for mental health conditions and related symptoms. The following research questions (RQs) were asked:

1. What are the differences between shortage and nonshortage areas regarding how people search for mental health symptoms?
2. How does the rural-urban divide interact with how people in shortage and nonshortage areas search for mental health symptoms?
3. How have patterns in searching for mental health symptoms in shortage and nonshortage areas changed over time, particularly given the onset of the COVID-19 pandemic?
4. How might search behavior concerning suicidal ideation differ between shortage and nonshortage areas?

Through our analysis, we demonstrate significant differences in how individuals in shortage and nonshortage areas articulate their experiences when seeking more information and resources regarding mental illness and health. We found that individuals tend to search for mental health less in shortage areas than in nonshortage areas. These results are consistent when accounting for the fact that shortage areas are predominantly rural. We also found that individuals in shortage areas tend to use broader (and often somatic) representations of mental illness when searching for resources, whereas individuals in nonshortage areas tend to search for more specific diagnoses and conditions that use clinical mental health language [32]. We also found distinct patterns regarding how people search for mental health and illness over time, including a specific look at suicidal ideation—a health issue with distinct prevalence and burden in shortage areas [33]. Overall, our approach demonstrates the viability of using symptom search data on the web to better understand the differences in how people understand and express their mental health experiences in resource-constrained areas.

## Methods

### Data

We leveraged data from the Google COVID-19 Search Trends symptoms data set [31]. Released during the COVID-19 pandemic and regularly updated, these data are “a publicly available dataset that shows aggregated, anonymized trends in Google searches for symptoms (and some related topics)” [34]. These data include searches related to 422 different symptoms, conditions, and diseases and not exclusively to mental health conditions. On their white page [32], Google described the process of aggregating and anonymizing health queries for each region in the data set. Using differential privacy, Google collected relative-to-area measures to search for queries related to the data set’s target terms. Statistical noise was added to obfuscate individual queries, especially in areas with low population where generalized statistics could be more invasive to specific individuals. As a show of face validity, these data have been used in past work to predict the rates of transmission and mortality of COVID-19 in the United States, both independently [35] and in conjunction with other data sources [36].

To scope our data set, we leveraged symptom search data at a biweekly level (the most granular available time span at the time of data collection) and at the county level for all counties in the United States. For our analysis, we analyzed data from 2017 to 2021.

We limited our analysis to all areas labeled as “Geographic Area” shortage and nonshortage areas by the US HRSA. This excluded smaller facilities that were population-based (such as “migrant farmworkers”) or facility-based (such as correctional facilities or “Indian Health Facilities”) [24]. In addition, to classify the relative rural or urban nature of different counties, we used the 3-tiered 2013 classification system delineating rural, micropolitan, and metropolitan areas used by the National Center for Health Statistics [37,38]. As a simplification, this classification system groups both moderately populated suburban areas and densely populated urban areas as being “metropolitan.” To make this analysis more specific and highlight the differences between the most rural and most urban counties, we also used the 6-tiered classification system [37] to examine the differences between the most rural counties and large “central” metropolitan areas (with populations of ≥1 million people). As some counties in the United States are classified as “partial shortage areas” [24], for a clearer comparison, we analyzed the differences between areas that were entirely classified as shortage areas or entirely not being shortage areas.

**Figure 1:**
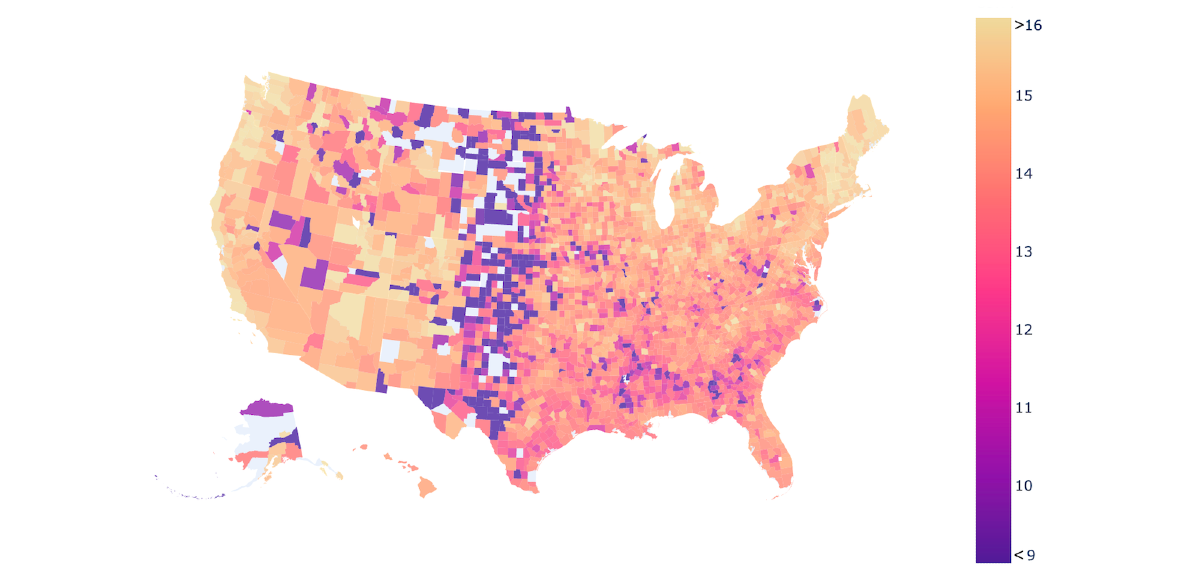
Map of US mental health searching percentage of total symptom search. Counties that tend toward purple have lower levels of mental health searching, whereas counties that have higher levels of mental health searching tend toward yellow. The maximum and minimum observed percentages of total searching that can be attributed to mental health can be seen in Table 1.

As a result of the robust process used to ensure that the search result data cannot be traced back to individual users [34], the Google COVID-19 Search Trends symptoms data set did not include data for counties that are extremely sparsely populated. In total, this included 105 counties (of the 3143 counties and county equivalents in the United States [39]), including 103 shortage area counties, 2 partial shortage counties, and 1 nonshortage county. In total, this is 3.37% of all counties or county equivalents (such as parishes or boroughs) in the United States. The specific counties that did not have available data, likely owing to extremely low population levels, are shown in Figure 1, colored in gray.

**Table 1:** Counties that search for mental health symptoms the most and the least.^a^

Rank	Counties with most mental health searching (%; area)	Counties with least mental health searching^b^ (%; area)
1	Uintah County, Utah (24.06%; shortage area)	Taliaferro County, Georgia (3.70%; shortage area)
2	Hampshire County, Massachusetts (17.74%; nonshortage area)	Irion County, Texas (4.27%; shortage area)
3	Oglala Lakota County, South Dakota (17.60%; shortage area)	Loving County, Texas (4.47%; shortage area)
4	Boulder County, Colorado (17.27%; shortage area)	Elk County, Kansas (4.57%; shortage area)
5	Todd County, North Dakota (17.24%; shortage area)	Harper County, Oklahoma (4.74%; shortage area)
6	Whitman County, Washington (17.19%; shortage area)	Clark County, South Dakota (4.84%; shortage area)
7	Douglas County, Kansas (17.17%; nonshortage area)	Hitchcock County, Nebraska (4.99%; shortage area)
8	Williamsburg City County, Virginia (17.16%; nonshortage area)	Quitman County, Georgia (4.99%; shortage area)
9	Tompkins County, New York (17.12%; shortage area)	Echols County, Georgia (5.03%; shortage area)
10	Monroe County, Indiana (17.09%; shortage area)	Eureka, Nevada (5.05%; partial shortage area)

^a^Percentages of mental health–related searches (out of total health-related searches) are in parentheses.

^b^Note that all counties with least mental health–related searches are either shortage or partial shortage areas.

### Identifying Mental Health Symptoms

As noted above, the Google COVID-19 Search Trends symptoms data set includes search topics that span both physical and mental health. To filter the particular topics that were primarily relevant to mental health, we began with a process of manual categorization by the 2 first coauthors (one of whom is a domain expert with nearly 6 years of experience in digital and global mental health). Each author selected search topics related to the conditions that were referenced in psychiatric manuals and resources. Comparing these independently generated lists resulted in a Cohen κ of 0.859, with an overall agreement of 97.6%. At this stage, we had identified 42 distinct topics pertaining specifically to mental health. We continued to refine and expand this list as described in the following paragraphs.

As discussed in previous research [40], broad and somatic symptoms can also be presentations of mental illness. As these were not captured in the initial pass of the 42 filtered symptoms above, we sought to expand our data set to include somatic symptoms that might be indicative of a mental illness. To do so, we identified symptoms that were most commonly searched on Google with the mental health diagnoses we had chosen. We leveraged Google Trends [41] to identify related search topics and queries associated with each of the 422 symptoms in our data set. We then probed these related topics and queries and created a list of symptoms and conditions that had connections to our manually generated list of 42 mental health symptoms and conditions. We included all symptoms that co-occurred with a manually selected mental health symptom or condition. As this process was likely to introduce noise in our data, we filtered out those associations that had a Google Trends relevance metric that was <2 (such as fecal incontinence or delayed onset muscle soreness). We decided to use 2 as our threshold after trying various threshold levels and optimizing the level that gave us the highest coverage with the least amount of noise in our list of symptoms. Using this strategy, we were able to discover relationships with symptoms that we had not selected manually but had important implications for mental health.

The affiliations output that we generated followed previous research in psychiatry. For example, there was a strong Google Trends signal between anxiety and depression, conforming to research in psychiatry that shows a high comorbidity between these 2 conditions [42,43]. There were also strong trends for other pairs that have been shown to have high comorbidities, such as insomnia and sleep apnea [44] or self-harm and physical scarring [45]. However, some of the strong affiliations were unexpected. For example, there was a strong association between confusion and urinary tract infections, likely attributable to the emergence of these symptoms together in older individuals potentially experiencing dementia [46].

Using these data on search affiliations from Google Trends, we were able to identify somatic symptom terminology that often co-occurred with mental health searches and were thus relevant to our analysis. To remove noise related to the COVID-19 pandemic, we manually removed any respiratory symptoms or any identified terms that were diagnosed as nonpsychiatric syndromes or broader diseases (as opposed to being solely symptoms). Our final list of 71 mental health–related search topics can be accessed in Multimedia Appendix 1.

### Analytic Approach

To protect the privacy of their users [34], the Google COVID-19 Search Trends symptoms data set provides aggregate normalized weights representing how often a symptom was searched in comparison to other symptoms rather than reporting raw search frequencies. As noted in the data description [31], because of the different scaling factors, these weights were not consistent across regions or timescales. Thus, to standardize our comparisons, we divided each symptom’s weight by the total summed weight of all symptoms for a given county and a given biweekly period to obtain an average percentage that an individual symptom had of all symptom searches. In our temporal analysis, we repeated this calculation but within a particular time frame, such as comparing the average percentage of a symptom between 2 given years, or in the case of our analysis, that of the influence of the pandemic on searching before and after the start of the COVID-19 pandemic. All comparisons were performed using a statistical 2-tailed *t* test. To eliminate the potential for a significant result occurring owing to chance because of the large number of symptoms being used as comparison points, we calculated the false discovery rate, hereinafter called the *Q* value, at a significance level of .05.

## Results

### Shortage Areas and Nonshortage Areas

We began our analysis by broadly examining the differences in searching for mental health symptoms between shortage and nonshortage areas (*RQ1*). As shown in Figure 1, searching for mental health symptoms was broadly associated with population density, similar to the association between mental health shortage area and population density [47]. More sparsely populated areas had lower levels of mental health symptom searching, whereas more populated areas had higher levels of mental health symptom searching.

Associations could also be observed in relation to race. As seen in Figure 1, noticeably lower levels of mental health symptom search could be seen in the Black Belt region of the American South. This region, originally called the Black Belt because of the dark soil typical of the area, is home to a higher-than-average number of Black individuals [48] as a result of free descendants of enslaved individuals who were economically forced to continue living in the same area as sharecroppers or tenant farmers [49]. As discussed by Pendse et al [50], the illness constructs used to evaluate mental health symptoms were primarily trained in White, Western, and often female populations. Therefore, it may be the case that these areas have comparable levels of mental health searching but may use cultural framings of distress [51,52] that are not typically associated with dominant framings of mental illness.

In addition, Table 1 shows the counties with the highest and lowest levels of mental health symptom searches. As shown, 3 of the top 10 counties with the highest rates of mental health symptom–related searching were nonshortage areas, whereas none of the areas with the lowest rates of mental health symptom–related searching were nonshortage areas. Nationally, individuals in shortage areas generally search for mental health symptoms less often than those in nonshortage areas. As noted in Table 2, on average, 13.42% (SD 2.24%) of searches in shortage areas were for mental health symptoms, compared with 15.06% (SD 0.96%) of searches in nonshortage areas.

When we break down the individual symptoms that make up our broader “mental health symptom” construct, we see some distinct patterns. Previous studies have shown that people living in rural areas define health issues broadly [53]. Indeed, broader and more general symptom categories associated with mental health tended to be searched significantly more often in shortage areas, specifically *anxiety* (mean 2.03%, SD 0.44%; *Q*), *depression* (mean 1.15%, SD 0.27%; *Q*), *fatigue* (mean 1.21%, SD 0.28%; *Q*), and *headache* (mean 1.03%, SD 0.23%; *Q*). The more clinical versions of these terms such as *major depressive disorder* or *generalized anxiety disorder* are less distinct, with *major depressive disorder* being searched more often in shortage areas (mean 1.01%, SD 0.25%), whereas *generalized anxiety disorder* is searched more often in nonshortage areas (mean 0.25%, SD 0.03%). However, the fact that clinical language around anxiety was searched for more in nonshortage areas may be representative of a greater level of mental health literacy, awareness of clinical framings of distress in nonshortage areas, or a previously known self-diagnosis, potentially stemming from access to a greater number of mental health professionals.

These differences were more distinct when searching for topics related to more specific symptom categories and mental health disorders. Individuals in nonshortage areas were significantly more likely to search for specific symptom categories and mental health disorders than individuals in shortage areas. For example, *binge eating* (mean 0.02%, SD 0.02%; *Q*), *psychosis* (mean 0.37%, SD 0.06%; *Q*), and *attention-deficit/hyperactivity disorder* (*ADHD*; mean 0.77%, SD 0.10%; *Q*) were all searched significantly more in nonshortage areas. These are clinically recognized in the Diagnostic Manual for Mental Disorders [54] and commonly appear in clinical interview instruments used by therapists, psychiatrists, and social workers [55]. The same is true of all other specific diagnoses other than *alcoholism*, which is searched more in nonshortage areas, but the search frequency is not statistically significantly distinct from that in shortage areas.

Pertinent to *RQ1***,** our results demonstrate that individuals in shortage areas were more likely than those in nonshortage areas to search for broader symptom categories, including those often associated with somatic symptom presentations of mental illness (such as headache or fatigue) [56,57]. These results point to a potentially strong relationship between the number of mental health practitioners in an area and how people come to understand and express their mental health in private and individual settings.

**Table 2:** Shortage versus nonshortage areas—*percentage of search*.^a,b^

			Shortage areas (%), mean (SD)		Nonshortage areas (%), mean (SD)	*Q* value
Overall average			13.42 (2.24)		15.06 (0.96)	1.26 × 10^–25^
**Broad symptoms**						
	Anxiety	2.03 (0.44)		1.8 (0.28)		6.36 × 10^–14^
	Depression	1.15 (0.27)		1.08 (0.11)		2.43 × 10^–4^
	Fatigue	1.21 (0.28)		1.05 (0.12)		5.64 × 10^–15^
	Headache	1.03 (0.23)		0.95 (0.07)		1.44 × 10^–7^
**Specific** **conditions**						
	*Alcoholism*	0.93 (0.29)		0.97 (0.14)		3.42 × 10^–2^
	*Attention-deficit/hyperactivity disorder*	0.73 (0.20)		0.77 (0.1)		8.80 × 10^–4^
	*Binge eating*	0.02 (0.02)		0.05 (0.02)		3.42 × 10^–52^
	*Compulsive behavior*	0.07 (0.06)		0.14 (0.03)		4.61 × 10^–71^
	*Dysphoria*	0.01 (0.02)		0.03 (0.02)		1.01 × 10^–59^
	*Generalized anxiety disorder*	0.18 (0.09)		0.25 (0.03)		7.76 × 10^–27^
	*Hypochondriasis*	0.008 (0.016)		0.017 (0.01)		4.57 × 10^–18^
	*Hypomania*	0.009 (0.02)		0.02 (0.01)		2.10 × 10^–29^
	Major depressive disorder	1.01 (0.25)		0.92 (0.09)		4.44 × 10^–8^
	*Manic disorder*	0.08 (0.06)		0.13 (0.03)		6.62 × 10^–48^
	*Mood disorder*	0.17 (0.09)		0.25 (0.04)		1.07 × 10^–28^
	*Psychosis*	0.30 (0.13)		0.37 (0.06)		3.80 × 10^–13^
	*Suicidal ideation*	0.05 (0.04)		0.10 (0.03)		2.57 × 10^–56^

^a^Percentages indicate the percentage of all health searches, with symptoms searched significantly more often in nonshortage areas (significance level of *Q*<.05) italicized. Nonitalicized symptoms were searched significantly more often in the shortage areas.

^b^The significance level of all *Q* values were <.0003, except for those of *alcoholism* (*Q*<.034) and *attention-deficit/hyperactivity disorder* (*Q*<.0009).

### The Rural-Urban-Metro Divide

To better understand whether the differences that we observed between shortage and nonshortage areas are influenced by rurality, we analyzed the differences between rural, urban, and metropolitan (areas with populations >1 million) shortage and nonshortage areas (*RQ2*). We began by examining the differences between rural shortage and rural nonshortage areas. We found that the searches for symptoms related to mental health are lower in rural shortage areas. We observed an average of 12.44% (SD 2.62%) mental health–related searches in rural shortage areas and of 14.71% (SD 2.44%) searches in rural nonshortage areas, consistent with what was observed in the broader shortage and nonshortage area comparison.

We found that broader symptoms were searched at similar rates between rural shortage and nonshortage areas. However, similar to our comparison between broad shortage and nonshortage areas, we found that though rural shortage areas search for *anxiety* more often, rural nonshortage areas search for the clinical term *generalized anxiety disorder* more often. Searches about *anxiety* (*Q*=.022) were observed significantly more frequently in rural shortage areas, accounting for an average of 2.24% (SD 0.49%) of searches related to mental health in rural shortage areas and of 1.87% (SD 0.29%) of searches in rural nonshortage areas. Searches for g*eneralized anxiety disorder* accounted for an average of 0.25% (SD 0.024%) of mental health–related searches in rural nonshortage areas and of 0.12% (SD 0.09%) of searches in rural shortage areas.

Next, consistent with our broad shortage and nonshortage area comparison, we find that more specific illness categories were searched significantly more often in rural nonshortage areas. *Compulsive behavior* (*Q*=1.16 × 10^–15^) and *psychosis* (*Q*=.002) were searched significantly more often in rural nonshortage areas (mean 0.121%, SD 0.049%; mean 0.383%, SD 0.12%, respectively) than in rural areas (mean 0.033%, SD 0.035%; mean 0.241%, SD 0.145%, respectively). Other specific conditions such as *manic disorder* or *mood disorder* that were searched significantly more often in rural nonshortage areas are shown in Table 3.

We then repeated this analysis for urban shortage and urban nonshortage areas to determine if the same patterns observed for rural shortage and nonshortage areas were preserved. Consistent with our past results, we noticed that individuals in urban nonshortage areas searched for mental health symptoms more often than individuals in urban shortage areas, with the percentage of all health searches being an average of 15.03% (SD 0.79%) in urban nonshortage areas and of 14.27% (SD 1.32%) in urban shortage areas.

We also observed that some broader and more somatic symptom categories, namely *fatigue* and *headache*, were higher in urban shortage areas than in urban nonshortage areas. However, broader psychiatric symptom categories were relatively similar, with no significant difference between urban shortage and nonshortage areas for searches related to *anxiety* (*Q*=.051) and *depression* (*Q*=.051), consistent with our rural analysis. Similar to the observations in our rural analysis, we found that specific conditions and disorders were searched significantly more often in urban shortage areas. As shown in Table 4, all specific conditions other than *major depressive disorder* were searched at higher rates in nonshortage areas. For example, *generalized anxiety disorder* (*Q*=5.69 × 10^–7^) was searched on an average of 0.23% (SD 0.07%) occasions in urban shortage areas and of 0.24% (SD 0.03%) in urban nonshortage areas. Similarly, *compulsive behavior* (*Q*=2.17 × 10^–20^) and *psychosis* (*Q*=.0014) were searched more often in urban nonshortage areas (mean 0.139%, SD 0.03%; mean 0.36%, SD 0.05%, respectively) than in urban shortage areas (mean 0.1%, SD 0.051%; mean 0.34%, SD 0.08%, respectively).

Analyzing our results in Tables 3 and 4 together, we see similar patterns when comparing shortage and nonshortage areas broadly. Individuals in shortage and nonshortage areas searched for broader symptom categories at roughly the same rates. However, individuals in nonshortage areas, rural or urban, relatively consistently searched for more specific conditions related to mental health. The number of mental health professionals that an area has might thus be an indication of how people in that area predominantly search for symptoms of mental illness, and this expresses how levels of mental health literacy relate to provider prevalence.

Subsequently, to investigate how rurality might influence how individuals in shortage areas express their symptoms, we examined differences in symptom searching in rural, urban, and metropolitan shortage areas. We found that rurality does influence how people frame their symptom searches. We discovered that the more rural a shortage area is, the lower their level of mental health searching is, with an average of 12.44% (SD 2.62%) of searches in rural shortage areas, 14.27% (SD 1.32%) of searches in urban shortage areas, and 15.11% (SD 0.70%) of searches in metropolitan shortage areas.

As shown in Table 5, when comparing rural and urban areas, we also found that searches for broader symptoms (such as *anxiety*, *depression*, *fatigue*, and *headache*) were significantly higher in rural shortage areas than in urban shortage areas. We also found that several specific diagnoses (such as *ADHD*, *generalized anxiety disorder*, and *psychosis* among others) are significantly more frequently searched in urban shortage areas, as shown in Table 5. This suggests that rurality is also a significant factor (along with the number of mental health professionals in an area) in influencing how people search for mental health resources on the web.

When comparing rural and metropolitan shortage areas, we observed similar patterns—searches were significantly higher for specific disorders and diagnoses in metropolitan shortage areas. However, we found that the overall degree of significance was smaller. For some of the broader symptoms, we found no statistically significant difference between the searches in rural shortage areas and metropolitan shortage areas (such as for *depression*, *major depressive disorder*, and *headache*). We did find that *anxiety* and *fatigue* were searched less frequently in metropolitan shortage areas than in rural shortage areas, consistent with our other results. Similarly, we did find that specific conditions were searched more often in metropolitan shortage areas than in rural shortage areas, as can be seen in Table 5.

Unique from other symptoms, we found no significant difference in the search rates of alcoholism among rural shortage areas, urban shortage areas, and metropolitan shortage areas (*Q*=.390; *Q*=.749). This confirms previous findings showing mixed results when attempting to examine differences in alcohol use disorders in urban and rural areas [58]. Taken together, these results enhance our understanding of the interaction between the rural and urban divide and shortage versus nonshortage discrepancies in mental health–related searching (*RQ2*).

**Table 3:** Rural shortage versus nonshortage areas—*percentage of search*.^a^

		Rural shortage areas (%), mean (SD)	Rural nonshortage areas (%), mean (SD)	*Q* value
Overall average		12.44 (2.62)	14.71 (2.44)	.007^b^
**Broad** **symptoms**				
	Anxiety^c^	2.24 (0.49)	1.87 (0.29)	.022^d^
	Depression	1.19 (0.35)	1.12 (0.26)	.550^e^
	Fatigue	1.28 (0.35)	1.06 (0.25)	.057^e^
	Headache	1.05 (0.30)	0.95 (0.15)	.343^e^
**Specific** **conditions**				
	Alcoholism^f^	0.93 (0.37)	1.18 (0.20)	.041^d^
	Attention-deficit/hyperactivity disorder	0.68 (0.25)	0.80 (0.24)	.192^e^
	Binge eating	0.013 (0.03)	0.019 (0.011)	.549^e^
	Compulsive behavior^f^	0.033 (0.035)	0.121 (0.049)	1.16 × 10^–15g^
	Dysphoria^f^	0.009 (0.02)	0.021 (0.02)	.042^d^
	Generalized anxiety disorder^f^	0.119 (0.09)	0.25 (0.024)	1.16 × 10^–6g^
	Hypochondriasis	0.008 (0.021)	0.003 (0.002)	.540^e^
	Hypomania	0.008 (0.020)	0.02 (0.03)	.330^e^
	Major depressive disorder	1.05 (0.33)	0.95 (0.20)	.441^e^
	Manic disorder^f^	0.040 (0.038)	0.13 (0.050)	2.13 × 10^–14g^
	Mood disorder^f^	0.117 (0.086)	0.255 (0.06)	1.48 × 10^–7g^
	Psychosis^f^	0.241 (0.145)	0.383 (0.12)	.002^b^
	Suicidal ideation^f^	0.024 (0.029)	0.088 (0.053)	1.91 × 10^–12g^

^a^Percentages indicate mental health searching out of all health searches.

^b^Significance level of *Q*<.01.

^c^Symptoms were searched significantly more in rural shortage areas.

^d^Significance level of *Q*<.05.

^e^Statistically insignificant, based on false discovery rate correction.

^f^Symptoms were searched significantly more in rural nonshortage areas.

^g^Significance level of *Q*<.001.

**Table 4:** Urban shortage versus nonshortage areas—*percentage of search*.^a^

			Urban shortage areas (%), mean (SD)		Urban nonshortage (%), mean (SD)		*Q* value
Overall average			14. (1.32)		15.03 (0.79)		5.71 × 10^–13b^
**Broad symptoms**							
	Anxiety	1.85 (0.32)		1.8 (0.3)		.051^c^	
	Depression	1.1 (0.17)		1.07 (0.09)		.051^c^	
	Fatigue^d^	1.13 (0.19)		1.05 (0.11)		1.54 × 10^–7b^	
	Headache^d^	1.01 (0.15)		0.95 (0.07)		1.29 × 10^–7b^	
**Specific conditions**							
	Alcoholism^e^	0.92 (0.19)		0.95 (0.12)		.033^f^	
	Attention-deficit/hyperactivity disorder^e^	0.76 (0.13)		0.77 (0.09)		.614^c^	
	Binge eating^e^	0.03 (0.02)		0.05 (0.02)		3.96 × 10^–19b^	
	Compulsive behavior^e^	0.1 (0.051)		0.139 (0.03)		2.17 × 10^–20b^	
	Dysphoria^e^	0.02 (0.018)		0.04 (0.02)		6.26 × 10^–19b^	
	Generalized anxiety disorder^e^	0.23 (0.07)		0.24 (0.03)		5.69 × 10^–7b^	
	Hypochondriasis^e^	0.01 (0.01)		0.02 (0.01)		9.97 × 10^–20b^	
	Hypomania^e^	0.01 (0.01)		0.02 (0.01)		2.01 × 10^-23b^	
	Major depressive disorder^d^	0.97 (0.16)		0.92 (0.08)		6.46 × 10^–6b^	
	Manic disorder^e^	0.11 (0.05)		0.13 (0.03)		8.23 × 10^–11b^	
	Mood disorder^e^	0.21 (0.07)		0.24 (0.04)		4.81 × 10^–8b^	
	Psychosis^e^	0.34 (0.08)		0.36 (0.05)		.0014^g^	
	Suicidal ideation^e^	0.07 (0.04)		0.09 (0.03)		5.21 × 10^–13b^	

^a^Percentages indicate mental health searching out of all health searches.

^b^Significance level of *Q*<.001.

^c^Statistically insignificant, based on false discovery rate correction.

^d^Symptoms searched significantly more in urban shortage areas.

^e^Symptoms searched significantly more in urban nonshortage areas.

^f^Significance level of *Q*<.05.

^g^Significance level of *Q*<.01.

**Table 5:** Percentage of searches in rural versus urban versus metro shortage areas.^a^

		Rural shortage areas (%), mean (SD)	Urban shortage areas (%), mean (SD)	Metro shortage areas (%), mean (SD)	Rural versus urban, *Q* value	Rural versus metro, *Q* value
Overall average		12.44 (2.62)	14.27 (1.32)	15.11 (0.70)	2.58 × 10^-68^^b^	.0003
**Broad symptoms**						
	Anxiety^c,d^	2.24 (0.49)	1.85 (0.32)	.97 (0.14)	1.08 × 10^–79b^	.0002^b^
	Depression^d^	1.19 (0.35)	1.1 (0.17)	1.05 (0.09)	5.89 × 10^–12b^	.16^e^
	Fatigue^c,d^	1.28 (0.35)	1.13 (0.19)	0.98 (0.03)	1.54 × 10^–26b^	.003^f^
	Headache^d^	1.05 (0.30)	1.01 (0.15)	0.91 (0.02)	.00023^b^	.083^e^
**Specific conditions**						
	Alcoholism	0.93 (0.37)	.92 (0.19)	0.97 (0.14)	.390^e^	.749^e^
	Attention-deficit/hyperactivity disorder^g^	0.68 (0.25)	0.76 (0.13)	0.78 (0.07)	7.26 × 10^–16b^	.193^e^
	Binge eating^g,h^	0.013 (0.03)	0.03 (0.02)	0.06 (0.01)	6.86 × 10^–61b^	1.18 × 10^–10b^
	Compulsive behavior^g,h^	0.033 (0.035)	0.1 (0.051)	0.19 (0.14)	1.93 × 10^–206b^	8.82 × 10^–42b^
	Dysphoria^d,g,h^	0.009 (0.02)	0.02 (0.018)	0.05 (0.01)	1.11 × 10^–48b^	8.83 × 10^–13b^
	Generalized anxiety disorder^g,h^	0.119 (0.09)	0.23 (0.07)	0.24 (0.007)	4.65 × 10^–134b^	2.56 × 10^–06b^
	Hypochondriasis^g,h^	0.008 (0.021)	0.01 (0.01)	0.03 (0.002)	.0009^b^	.0017^f^
	Hypomania^g,h^	0.008 (0.020)	0.01 (0.01)	0.03 (0.005)	3.29 × 10^–08b^	7.36 × 10^–05b^
	Major depressive disorder^d^	1.05 (0.33)	0.97 (0.16)	0.88 (0.06)	2.10 × 10^–09b^	.074^e^
	Manic disorder^g,h^	0.040 (0.038)	0.11 (0.05)	0.14 (0.01)	1.37 × 10^–208b^	2.07 × 10^–20b^
	Mood disorder^g,h^	0.117 (0.086)	0.21 (0.07)	0.24 (0.018)	1.36 × 10^–132b^	5.87 × 10^–07b^
	Psychosis^g,h^	0.241 (0.145)	0.34 (0.08)	0.38 (0.03)	1.04 × 10^–66b^	.0015^f^
	Suicidal ideation^g,h^	0.024 (0.029)	0.07 (0.04)	0.11 (0.02)	9.11 × 10^–172b^	8.78 × 10^–22b^

^a^Percentages indicate the percentage of all health searches, with SDs in parentheses.

^b^Significance level of *Q*<.001.

^c^Symptoms searched more often in rural shortage areas than in metro shortage areas.

^d^Symptoms searched more often in urban shortage areas.

^e^Statistically insignificant, based on false discovery rate correction.

^f^Significance level of *Q*<.01.

^g^Symptoms searched significantly more often in rural shortage areas than in urban shortage areas.

^h^Symptoms searched more often in metropolitan shortage areas.

### Temporal Analysis of Differences and the COVID-19 Pandemic

**Figure 2:**
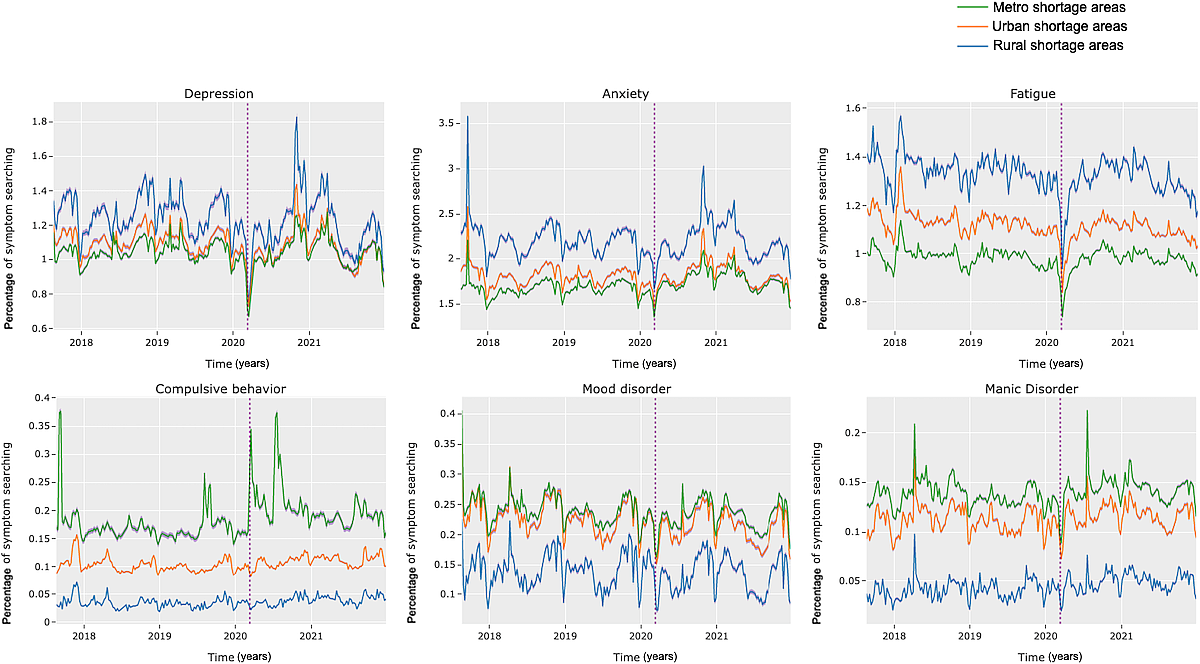
Symptom search distributions for several mental health symptoms in shortage areas. Vertical line represents March 11, 2020, the day that the World Health Organization (WHO) declared COVID-19 a global pandemic [61]. CIs represented in transparent purple (the CIs tightly follow the mean). Note that in the broader terms on the top row, rural shortage areas search at a higher rate than other types of areas. For the more particular terms on the bottom row, rural shortage areas search at significantly lower rates.

**Figure 3:**
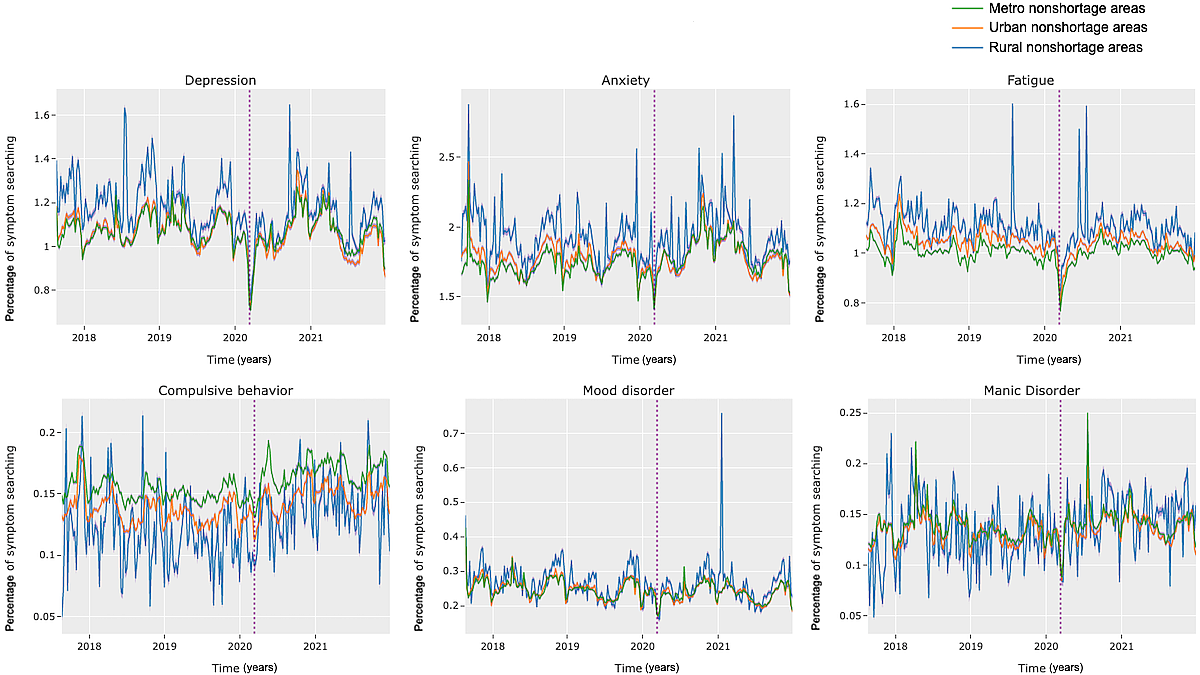
Symptom search distributions for several mental health symptoms in nonshortage areas. Vertical line represents March 11, 2020, the day that the World Health Organization (WHO) declared COVID-19 a global pandemic [61]. CIs represented in transparent purple (the CIs tightly follow the mean).

**Figure 4:**
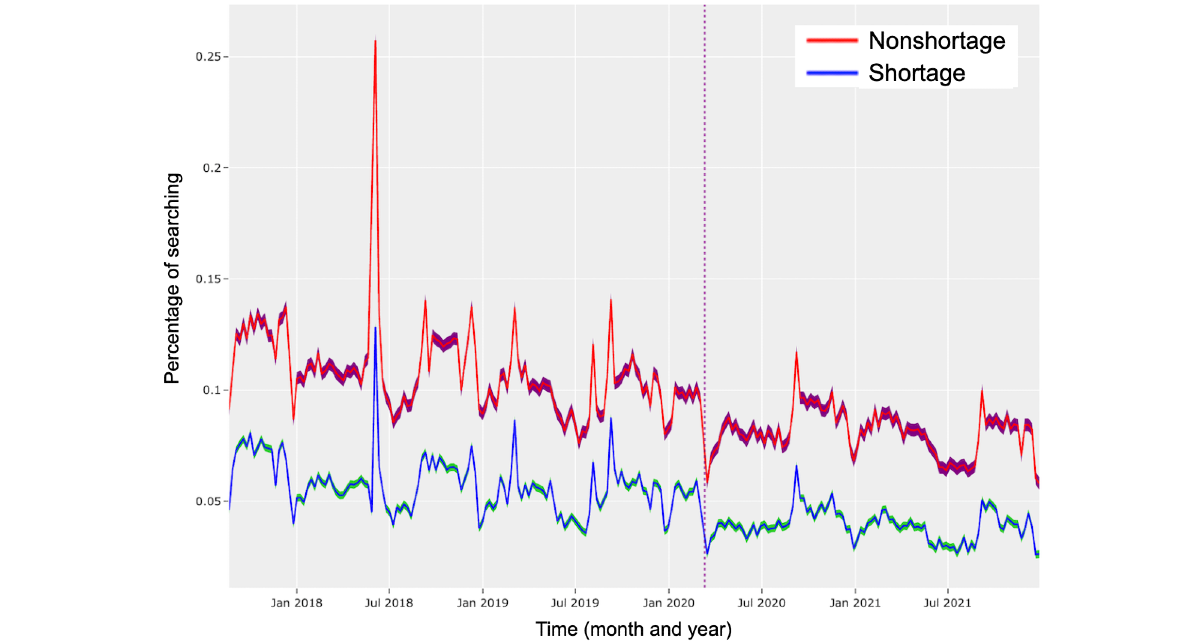
Symptom search distributions for suicidal ideation. Vertical line represents March 11, 2020, the day that the World Health Organization (WHO) declared COVID-19 a global pandemic [61]. Note that symptom searching in both shortage and nonshortage areas trends downward. Also note that shortage areas (with higher overall suicide rates) have lower levels of searching about suicidal ideation. Jan: January; Jul: July.

In this section, we examine the differences in search behavior regarding mental health in shortage and nonshortage areas over time (*RQ3*). As demonstrated in Figures 2 and 3, though trends tended to be similar among rural, urban, and metropolitan areas, the relative gaps (in searching) between areas tended to remain consistent over time in both shortage and nonshortage areas. The similarity in these dynamics suggests that symptom searching in rural, urban, and metropolitan areas is likely to be similarly affected by external factors or events, though to different magnitudes. In addition, as demonstrated in Figure 4, using the example of searches related to *suicidal ideation*, we also noticed that gaps between shortage and nonshortage areas also remained consistent over time.

One trend we want to draw particular attention to is the decrease in searching for mental health symptoms at the onset of the COVID-19 pandemic (March 11, 2020 [60]). As indicated by the vertical lines in Figures 2, 3, and 4, at the onset of the pandemic, individuals in the shortage and nonshortage areas began to search for mental health significantly less often. This likely demonstrates that mental health took a backseat as individuals searched for health information related to COVID. Over time, levels of mental health symptom searching stabilized to similar levels in both shortage and nonshortage areas, a finding that has also been observed in other contexts regarding the expression of psychosocial concerns during the pandemic [61]. Researchers attribute this plateauing effect to habituation to a “new normal,” given the protracted nature of the pandemic [61].

Although these overall trends tend to be similar, we did observe some differences in mental health symptom searching before and after the pandemic’s onset. As shown in Table 6 for shortage areas and in Table 7 for nonshortage areas, searches for *headache*, *ADHD*, *compulsive behavior*, *dysphoria*, *hypochondriasis*, and *manic disorder* significantly increased after the start of the COVID-19 pandemic in both shortage and nonshortage areas. In addition, in both the shortage and nonshortage areas, searches for *depression*, *fatigue*, *alcoholism*, *major depressive disorder*, *mood disorder,* and *suicidal ideation* significantly declined. However, it has been observed that overall, alcohol use [59] and depressive symptoms [62,63] increased during the pandemic. A demonstrated lack of engagement with web-based resources may thus be an indication of a lack of engagement with offline resources.

**Table 6:** *Percentage of search* in shortage areas during pre–COVID-19 pandemic versus post–COVID-19 pandemic.^a^

			Pre–COVID-19 pandemic (%), mean (SD)		Post–COVID-19 pandemic (%), mean (SD)		*Q* value
Overall average			13.65 (0.70)		13.89 (0.97)		.031^b^
**Broad symptoms**							
	Anxiety	2.006 (0.153)		2.021 (0.181)		.505^c^	
	Depression^d^	1.186 (0.091)		1.136 (0.147)		.0023^e^	
	Fatigue^d^	1.242 (0.057)		1.203 (0.065)		3.95 × 10^–6f^	
	Headache^g^	1.047 (0.047)		1.069 (0.055)		.0026^e^	
**Specific conditions**							
	Alcoholism^d^	0.957 (0.058)		0.928 (0.07)		.0017^e^	
	Attention-deficit/hyperactivity disorder^g^	0.729 (0.109)		0.788 (0.147)		.0010^e^	
	Binge eating	0.022 (0.005)		0.023 (0.005)		.303^c^	
	Compulsive behavior^g^	0.068 (0.011)		0.074 (0.009)		3.49 × 10^–6f^	
	Dysphoria^g^	0.012 (0.003)		0.017 (0.004)		8.26 × 10^–23f^	
	Generalized anxiety disorder	0.183 (0.019)		0.179 (0.023)		.354^c^	
	Hypochondriasis^g^	0.007 (0.002)		0.008 (0.002)		.008^e^	
	Hypomania	0.009 (0.003)		0.010 (0.002)		.048^b^	
	Major depressive disorder^d^	1.066 (0.088)		1.008 (0.098)		1.36 × 10^–5f^	
	Manic disorder^g^	0.076 (0.012)		0.082 (0.011)		9.64 × 10^–5f^	
	Mood disorder^d^	0.184 (0.029)		0.170 (0.029)		5.36 × 10^–4f^	
	Psychosis^g^	0.310 (0.036)		0.322 (0.035)		.020^b^	
	Suicidal ideation^d^	0.057 (0.013)		0.038 (0.007)		3.48 × 10^–27f^	

^a^Percentages indicate percentage of all health searching.

^b^Significance level of *Q*<.05.

^c^Statistically insignificant, based on false discovery rate correction.

^d^Symptoms searched significantly more before March 15.

^e^Significance level of *Q*<.01.

^f^Significance level of *Q*<.001.

^g^Symptoms searched significantly more after March 15.

**Table 7:** *Percentage of search* in nonshortage areas during pre–COVID-19 pandemic versus post–COVID-19 pandemic.^a^

		Pre–COVID-19 pandemic (%), mean (SD)	Post–COVID-19 pandemic (%), mean (SD)	*Q* value
Overall average		14.985 (0.64)	15.234 (0.86)	.02^b^
**Broad symptoms**				
	Anxiety^c^	1.772 (0.116)	1.808 (0.135)	.038^b^
	Depression^d^	1.088 (0.106)	1.051 (0.106)	.0031^e^
	Fatigue^d^	1.056 (0.050)	1.032 (0.050)	1.98 × 10^–4f^
	Headache^c^	0.936 (0.0004)	0.955 (0.0004)	6.38 × 10^–4f^
**Specific conditions**				
	Alcoholism^d^	0.977 (0.051)	0.943 (0.068)	5.03 × 10^–5f^
	Attention-deficit/hyperactivity disorder^c^	0.747 (0.084)	0.804 (0.124)	8.23 × 10^–5f^
	Binge eating^c^	0.049 (0.008)	0.052 (0.008)	.0042^e^
	Compulsive behavior^c^	0.136 (0.012)	0.148 (0.012)	1.01 × 10^–11f^
	Dysphoria^c^	0.030 (0.005)	0.043 (0.008)	2.42 × 10^–33f^
	Generalized anxiety disorder^d^	0.251 (0.019)	0.241 (0.020)	.002^b^
	Hypochondriasis^c^	0.017 (0.003)	0.020 (0.004)	2.18 × 10^–9f^
	Hypomania^c^	0.022 (0.005)	0.024 (0.003)	9.06 × 10^–5f^
	Major depressive disorder^d^	0.929 (0.069)	0.895 (0.079)	8.06 × 10^–4f^
	Manic disorder^c^	0.133 (0.014)	0.139 (0.015)	.002^e^
	Mood disorder^d^	0.254 (0.031)	0.237 (0.026)	3.96 × 10^–5f^
	Psychosis	0.369 (0.031)	0.368 (0.028)	.760^g^
	Suicidal ideation^d^	0.108 (0.021)	0.081 (0.010)	4.77 × 10^–24f^

^a^Percentages indicate percentage of all health searching.

^b^Significance level of *Q*<.05.

^c^Symptoms searched significantly more after March 15.

^d^Symptoms searched significantly more before March 15.

^e^Significance level of *Q*<.01.

^f^Significance level of *Q*<.001.

^g^Statistically insignificant, based on false discovery rate correction.

### Suicidal Ideation

In this section, we analyze the trends in suicidal ideation between shortage and nonshortage areas (*RQ4*). Previous research by Ku et al [33] found that the rates of death by suicide are consistently higher in shortage areas than those in nonshortage areas. In addition, Ku et al also found that the association between having a higher suicide rate and being in a shortage area has grown over time.

Overall, searches for suicidal ideation tended to be at an average of 0.05% (SD 0.04%) of all mental health–related searches in shortage areas and of 0.10% (SD 0.03%) of all the searches in nonshortage areas. This pattern was also observed in rural and urban shortage areas, with averages of 0.024% (SD 0.029%) of searches in rural shortage areas, 0.088% (SD 0.053%) of searches in rural nonshortage areas, 0.07% (SD 0.04%) of searches in urban shortage areas, and 0.09% (SD 0.03%) of searches in urban nonshortage areas. Rurality was also significantly related to how people search about suicide in shortage areas—individuals in rural shortage areas (mean 0.024%, SD 0.029%) tended to search for suicide at lower rates than individuals in urban shortage areas (mean 0.07%, SD 0.03%; *Q*=9.11 x 10^-172^) and even more so than those in metropolitan shortage areas (mean 0.11%, SD 0.03%; *Q*=8.78 x 10^-22^). Although the rates of suicide are consistently known to be higher in shortage [33] and rural areas [64], we observed that the rates of *searching* for suicidal ideation were actually lower in shortage areas (*Q*<.0003), and more so when a shortage area was rural (*Q*<2 × 10^–12^).

In addition, previous work has shown that the rates of suicide have increased over time (from 1999 to 2016), with the steepest rises in rural areas [65]. However, considering the rates of searching, we actually observed that the rates of searching for suicidal ideation slowly trending downward over time. We conjecture that this discrepancy might be representative of the fact that people who search for resources receive more support (via internet resources) and are thus less likely to die by suicide.

## Discussion

### Contextualizing Findings in Previous Research: Implications for Search Engines

We observed that search topics pertaining to specific clinical language regarding mental health were searched more often in nonshortage areas than in shortage areas, regardless of whether the shortage area was rural or urban. This finding allowed us to uncover the relationship between the number (or availability) of mental health professionals in an area and the language that people use to describe their experiences of distress (via search data). Having more specialized language to express mental health status can be key to obtaining more specific treatment options. However, individuals in shortage areas tend to use this specific language less frequently.

Search engines can help close this inequity in care by referring individuals to specialized resources regarding mental health, even when the symptom categories being searched for are broader. For example, the results in a search for “headache” might include emotional support materials and information about mental health conditions alongside broader information about the different causes of or remedies for a headache. Doing so might also moderate the potential for search engine results to escalate anxieties related to the diagnosis for a user, especially from a shortage area. White and Horvitz [66] described how the use of search engines to gain diagnostic understanding of illnesses by individuals with limited medical training may sometimes result in unfounded escalation of concerns regarding common symptomatology. The presence of emotional support and mental health resources alongside traditional health information could provide individuals with language and resources that are more in line with their experience of distress and serve to de-escalate concerns about symptoms that are likely to be nonlethal.

The need for an equitable design of search engines that attends to the diverse mental health needs of underserved populations is underscored by the fact that technology-mediated systems may be the most accessible form of care or resources available to communities in need [67]. Therefore, in addition to the search engines being intentional about the type of support resources to be directed to underserved mental health search users, we also emphasize the need to consider alternative ways of optimizing matching and personalization in search. In particular, while search engines do consider geolocation as one of many variables in tailoring search results [68], our findings show that given the nature of searches in shortage and nonshortage areas, personalization should consider social determinants of health and structural inequities in care experienced by these communities. For instance, search engines, which are equipped with the insights gained from this work, could strive to provide more educational pointers that enhance understanding of one’s experience as a result of broad symptom searching in shortage areas. Similarly, as users in nonshortage areas tend to use more clinical terms to seek information on mental health, search engine algorithms may prioritize those results that provide advice complementary to formal treatment.

We note that search technologies encode certain values about what sort of content is “important” or “authoritative” [69], and previous work has discovered that a lack of consideration of equities can result in amplified biases against minoritized identities [70,71]. Although we do not discover the underlying intent or offline context of specific search behaviors, when catering to mental health–related search queries, these values would need to be punctuated with a deeper understanding of the types of searches in shortage and nonshortage areas. More specifically, we argue that to fulfill the potential of serving as an algorithmically mediated care resource, search engine design will need to decouple itself from the biases encoded in web-based advertising delivery. For instance, it has been demonstrated that search results for Black-identifying first names are associated with more advertisements for public record searches (eg, arrest records), in contrast to those for White-identifying first names [72]. Search engines would need to pay careful attention to ensure that queries for broader mental health symptoms, as observed more often in shortage areas, provide empirically grounded results and not advertisements for unverified treatments.

### Contextualizing Findings in Previous Research: Implications for Public Health

Our approach demonstrates the potential of using search engine log data to better understand emerging symptom presentations and the use of care resources, expanding earlier investigations that found individuals using search engines to gather new information and resources for their health [30]. Therefore, search engine log data can be used to identify emerging symptom presentations by analyzing what searches co-occur with mental health symptoms. Moreover, from previous work, we already know that interactions, which are mediated by search engine ranking algorithms and their corresponding interfaces, can have tangible impacts on how individuals understand, describe, and present their symptoms when speaking to clinicians [73,74]. Thus, search log data, such as the one used here, could also be used to identify emerging shortage areas or nonshortage areas, even in areas with less reliable offline data. Areas in which individuals seem to tend toward searching broader symptom categories could be flagged as potential shortage areas, whereas areas with individuals who seem to tend toward searching more specific diagnoses could be flagged as potential nonshortage areas. Combined with traditional metrics from the HRSA, this information could be used in new programs to incentivize mental health professionals to work in less covered areas. Search engines could differentiate between resources based on the shortage status and corresponding mental health literacy rate that are inferred from a region’s search queries. This could be particularly helpful in the case of suicidal ideation. We find that though rates of suicide are higher in shortage areas and on the increase, broadly, rates of *searching* for suicidal ideation are lower in shortage areas and on the decline overall. Providing additional resources to support someone experiencing suicidal ideation, even when queries may not be specific to suicidal ideation, may lead individuals to discover resources to which they may not have otherwise been exposed (such as helplines, warmlines, or peer respite centers). This finding also underscores the need to augment public health informational campaigns that may be specifically targeted to underserved communities, as stigma and limited literacy around suicidal risk may be underlying factors driving lower search for information regarding suicide in shortage areas.

### Ethical Implications and Future Work

On the basis of our results, we believe and argue that search engine algorithm designers have an ethical imperative to use US public health information (such as whether an area is a shortage area) and apparent search trends to offer mental health resources more readily. However, as Pendse et al [50] discussed, we also consider the fact that the form of resources recommended might influence how people come to think of their mental health state. Privileging biomedical approaches to health might be counterproductive or crowd out minoritized forms of care. An alternative approach (such as an issue-specific support group or online forum) might be more in line with how an individual understands their distress. We contend that search engine algorithm designers should ensure that resources offered to individuals who appear to be in distress in prioritized medical information cards (such as those from Google’s Knowledge Panel [75]) are diverse, such as broader resources for stress, warmlines, peer support, and other forms of care that may not fit a traditional biomedical lens, as Pendse et al [50] recommend. Similarly, we are cognizant of the potential for analyses of search engine data to be used to expose users’ personal information without consent, particularly the information of those living in geographically sparse or underserved areas. We appreciate the use of several privacy-preserving mechanisms in the Google COVID-19 Search Trends data set [34].

Our work highlights the importance of better understanding the differences between how individuals in shortage and nonshortage areas perceive their experience of distress and express it to others. However, the data we analyzed were anonymized metadata and did not include other axes of oppression [76,77] that have an influence on expressions of mental illness, such as race, gender identity, sexual orientation, class, caste, ethnicity, or nationality [50]. Future work must qualitatively investigate the specific idioms of distress [78] and explanatory models [79] that underlie these search queries. Our work is limited to resource-constrained areas in the United States, and future work could explore how resource constraints interact with broader mental health symptom–related searches in other countries.

### Conclusions

Technology-mediated tools for mental health support (and the algorithms that underlie them) can often be the first point of contact with any form of mental health resources, particularly for those in underserved groups. The diverse ways in which individuals understand and express their mental illness thus have a direct influence on both what resources are recommended and what resources are proactively excluded. In this study, we investigated mental health symptom searching among one such underserved group or individuals in the US MHPSAs. We demonstrated strong differences between how individuals in areas with fewer mental health professionals search about their mental health, with clear implications for algorithm design and health equity. We leveraged this analysis to discuss how search engine algorithm designers might be conscientious of the role that structural factors play in expressions of distress and how they can design search engine algorithms and interfaces to close gaps in care.

## Appendix

Multimedia Appendix 1 List of mental health–related search topics.

## Abbreviations

ADHD: attention-deficit/hyperactivity disorder
HRSA: Health Resources and Services Administration
MHPSA: Mental Health Professional Shortage Area
RQ: research question
